# New advances in anti‐microbials: An annotated selection of World Wide Web sites relevant to the topics in *microbial biotechnology*

**DOI:** 10.1111/1751-7915.14019

**Published:** 2022-02-24

**Authors:** Lawrence P. Wackett

**Affiliations:** ^1^ Department of Biochemistry, Molecular Biology & Biophysics BioTechnology Institute University of Minnesota St Paul MN 55108 USA

## Fighting antibiotic resistance: CDC

### https://www.cdc.gov/drugresistance/solutions‐initiative/innovations‐to‐slow‐AR.html

This website for the Center for Disease Control highlights their investments in responding to antibiotic resistance. One interesting resource is a large strain collection of resistant isolates that can be accessed for research purposes.

## Pipeline of antibiotics in development

### https://www.pewtrusts.org/en/research‐and‐analysis/issue‐briefs/2021/03/tracking‐the‐global‐pipeline‐of‐antibiotics‐in‐development

This page of the Pew Trust lists provides links to ongoing projects for new antibiotic development.

## Advances in antimicrobial innovation

### https://royalsociety.org/science‐events‐and‐lectures/2021/07/tof‐amr/

This page from the Royal Society is for a scientific meeting on the subject. A link is provided to a report on the meeting.

## Artificial intelligence for developing new antibiotics

### https://news.mit.edu/2020/artificial‐intelligence‐identifies‐new‐antibiotic‐0220

This MIT News page describes the development of a model trained on known antimicrobial substances to predict the efficacy of new compounds from a chemical library. Subsequent testing showed the effectiveness of the approach.

## Antimicrobial resistance: WHO

### https://www.who.int/news‐room/fact‐sheets/detail/antimicrobial‐resistance

This page from the World Health Organization provides a good overview of global concerns and research regarding antimicrobial resistance.

## Biosynthetic gene clusters: MIBiG 2.0

### https://pubmed.ncbi.nlm.nih.gov/31612915/

MIBiG is a data repository for natural product gene clusters of known functions. This serves as an important tool for uncovering the functions in new gene clusters that will provide the basis for future antimicrobials.

## Recent advances in antimicrobial nanodrugs

### https://www.mdpi.com/journal/pharmaceuticals/special_issues/Antimicrobial_Nanodrugs

This is the webpage for a special issue of the journal *Pharmaceuticals* that is scheduled to be published in this coming year, 2022.

## Natural products in drug discovery

### https://www.nature.com/articles/s41573‐020‐00114‐z

This recent review article summarizes technical advances that are promoting new natural product drug discovery, including antimicrobials.

## The International Natural Product Sciences Taskforce

### http://inpst.net/

This page represents a large group of scientists working in the area of natural products and has many links to publications and resources.

## *Nature* review on new antibiotic development

### https://www.nature.com/articles/s41570‐021‐00313‐1

This recent review highlights new developments in generating pipelines for new antibiotic discovery and implementation.

